# Stem cell models of human embryo implantation and trophoblast invasion

**DOI:** 10.1016/j.gde.2025.102357

**Published:** 2025-05-20

**Authors:** Peiheng Liu, Serene Mattis, Thorold W Theunissen

**Affiliations:** 1 Department of Developmental Biology and Center of Regenerative Medicine, Washington University School of Medicine, St. Louis, MO 63110, USA; 2 Department of Biomedical Engineering, Washington University in St. Louis, St. Louis, MO 63130, USA

## Abstract

Stem cell–based embryo models have taken the scientific community by storm as they enable investigation of previously inaccessible stages of human development. Here, we discuss how stem cell–based embryo and placenta models can shine a light on two elusive and intertwined aspects of human development that are critical for successful pregnancy: the implantation of the blastocyst into the endometrium and the subsequent invasion of placental villi deep inside the maternal tissues. Both of these processes are mediated by the trophoblast lineage, which is specified in the preimplantation embryo and can be modeled using naïve pluripotent stem cells. We review how embryo and placenta models built from naïve stem cells can be leveraged to obtain mechanistic insights into human implantation and trophoblast invasion.

## Introduction

**Figure 1:**
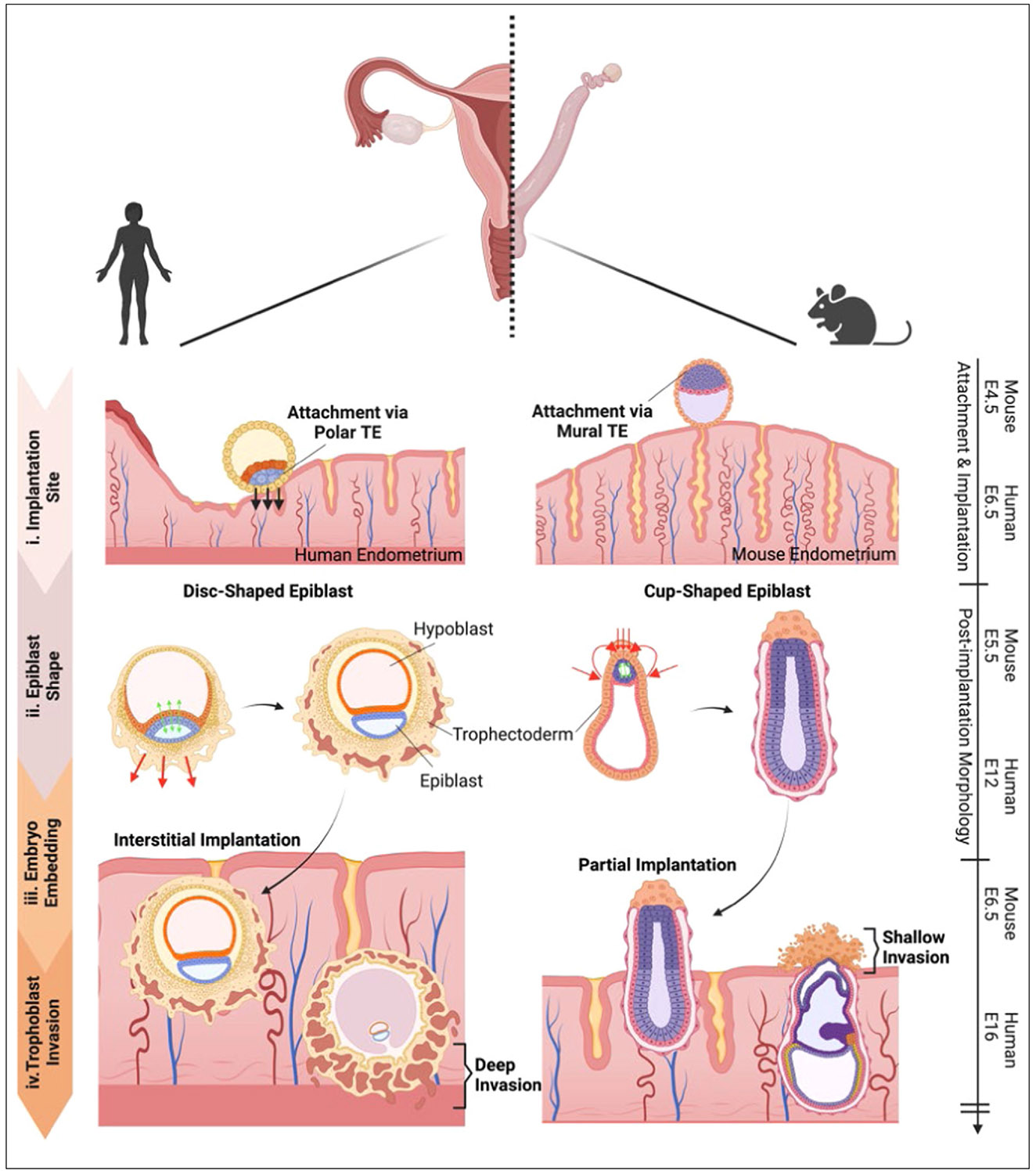
Major differences between mouse and human implantation. This figure contrasts human and mouse implantation processes, highlighting significant differences in TE attachment, epiblast morphology, embryo embedding, and trophoblast invasion depth. These differences illustrate species-specific adaptations during early development and highlight the need for human-specific model systems of embryo implantation and trophoblast invasion.

The early stages of human development have remained a scientific black box due to ethical and practical restrictions on research involving human embryos. A historical collection of serially sectioned human embryos, commonly referred to as the Carnegie Collection, provided an initial window into the major morphological changes of human embryos during the first 8 weeks of development [1,2]. These snapshots of early human development highlighted several important differences with the mouse (Figure 1): (i) human embryos implant via the polar region of trophectoderm (TE), the outer layer of the blastocyst nearest the inner cell mass (ICM), while mouse blastocysts implant via the opposite side; (ii) the human epiblast, the founder tissue of the embryo proper, forms a disc-shaped epithelium after implantation, while the mouse epiblast forms a cup-shaped epithelium; (iii) the human embryo becomes fully embedded inside the uterus, while the mouse embryo only implants partially; (iv) invasion by trophoblasts, which emerge from the TE and constitute the major epithelial cell type in the placenta, is significantly more limited and shallow in mice. In contrast, human trophoblasts invade deep into the uterine tissue, reaching even into the myometrium, the muscular middle layer of the uterine wall. Given these and other significant differences, it has been challenging to establish effective mouse models for human implantation and placental development.

Human implantation and placental development are not only of fundamental interest to developmental biologists but also hold important value for understanding the etiology of pregnancy-related diseases. Complications during the implantation process are a major cause of *in vitro* fertilization failure and early miscarriage [3]. It is also a major bottleneck for a healthy pregnancy, as only 30% of human embryos undergo successful implantation. Preeclampsia, a dangerous hypertensive disorder that affects 2–8% of pregnancies worldwide, is thought to be a result of shallow trophoblast invasion into the maternal tissues during the first trimester of pregnancy [4]. Primary placental cultures and immortalized trophoblast cell lines have been isolated but fail to recapitulate trophoblast differentiation from a progenitor state [5-7]. Consequently, there remains a large unmet need for human-specific model systems of blastocyst implantation, trophoblast invasion, and their pathologies.

Recent breakthroughs in the generation of stem cell–based embryo and placenta models offer a viable alternative to human embryos or animal models in interrogating early human development. Of particular relevance to modeling human implantation are blastocyst-like structures (also known as ‘blastoids’) generated from naïve human pluripotent stem cells (hPSCs), which are a primitive type of stem cell that resembles pluripotent cells in the human blastocyst [8-10]. Blastoids consist of an outer TE layer and an ICM and model the early stages of human postimplantation development when transferred to appropriate matrices [11,12]. To model human placental development, researchers have turned to human trophoblast stem cells (hTSCs) and trophoblast organoids (TOs), which can be derived either from primary tissues [13-15] or naïve hPSCs [16-19]. In this review, we discuss how human embryo and placenta models can shine a light on the elusive processes of blastocyst implantation and trophoblast invasion.

## Human blastocyst implantation

### Current understanding and model systems

Implantation is a critical phase in early human development during which the blastocyst, formed approximately 1 week after fertilization, attaches to and invades the endometrium, the outer lining of the uterus. This process is essential for establishing successful pregnancy and is marked by specific stages. Initially, the blastocyst undergoes *apposition*, a temporary interaction with the endometrium. This is followed by *adhesion*, where the TE establishes firmer contact with the uterine lining. Finally, in the *invasion* stage, trophoblast cells penetrate into the endometrium, reaching even into the deeper-lying myometrium. During this stage, trophoblasts invade and remodel spiral arteries, allowing nutrient and gas exchange between mother and embryo. Mouse models have uncovered an intricate maternal–fetal dialog involving adhesion molecules, lipid mediators, transcription factors, and the extracellular matrix (ECM) [20,21]. A classical example is the interaction between epidermal growth factor (EGF), a protein expressed in the uterine luminal epithelium, and the receptors ErbB1 and ErbB4 on the blastocyst surface, which is critical for timely blastocyst attachment [22,23]. However, given the numerous differences between mouse and human implantation highlighted above, human stem cell–based embryo models are being developed to fill in the gaps.

A suitable blastocyst surrogate is essential for modeling human implantation. Inspired by mouse blastoids [24], three groups reported human blastoid generation from naïve hPSCs in 2021–2022 [8-10]. Unlike mouse blastoids, human blastoids were formed solely from naïve hPSCs, which have broad developmental potential. Despite differences in culture and aggregation conditions, all blastoids exhibited key morphological features: an outer TE layer and an ICM containing epiblast and primitive endoderm. Single-cell RNA sequencing (scRNA-seq) confirmed their transcriptional fidelity, with Kagawa et al. and Yanagida et al. showing over 97% of cells matching blastocyst-stage embryos. These two studies generated blastoids from naïve cells derived with the PXGL method [8,10]. Yu et al., using naïve cells derived using an alternative culture formulation, called 5iLA, reported more undefined cell types in blastoids [9], though later optimizations improved lineage fidelity [11,12]. These studies highlight human blastoids as transcriptionally relevant models of preimplantation development.

When attached to tissue culture plastic, human embryos can initiate early stages of postimplantation development in the absence of maternal tissues [25,26]. The ability to progress through key developmental milestones without the uterine environment has inspired researchers to investigate whether human blastoids share this capacity. Yu et al. and Yanagida et al. demonstrated that blastoids could mimic peri-implantation structures, showing early signs of epiblast lumen formation and TE remodeling [9,10]. Karvas et al. extended this by culturing blastoids on a 3D matrix, revealing hallmarks of early postimplantation development, including epiblast lumenogenesis, trophoblast expansion, and asymmetric expression of primitive streak marker TBXT by day 21. scRNA-seq analysis showed transcriptome profiles resembling the Carnegie Stage 7 human gastrula. While amnion and hypoblast formation were delayed, these findings highlight blastoids’ ability to model early human development from preimplantation to early gastrulation [12]. A meta-analysis confirmed that 3D-cultured blastoids exhibit a broad range of trophoblast cell types, modeling human trophoblast differentiation across implantation [27].

Importantly, the success of implantation is an interactive process between the blastocyst and maternal cell types within the uterus. The period of uterine receptivity for implantation is limited and referred to as the ‘implantation window’, which is regulated by ovarian steroid hormones [20]. Turco et al. pioneered the establishment of human endometrial organoids by embedding glandular epithelial cells into an ECM [28]. These organoids maintain epithelial polarity and respond to reproductive hormones, which allows them to simulate physiological conditions of the endometrium, including hormonal cycles and decidualization, a process that prepares the endometrium for pregnancy. Similarly, Boretto et al. developed endometrial organoids by embedding dissociated endometrial fragments in Matrigel and optimizing conditions with WNT activation [29]. Their model also responds to estrogen and progesterone, mirroring cyclical endometrial changes and supporting longterm culture.

Recent studies have made strides in replicating the dynamic interactions between the blastocyst and endometrial tissues by co-culturing human blastoids with endometrial cell models. Kagawa et al. developed an *in vitro* implantation model by combining human blastoids with an open-face endometrial layer [8]. By seeding blastoids onto this two-dimensional layer of endometrial epithelial cells, they observed that blastoids specifically attached to receptive, hormone-stimulated endometrial cells through the polar TE, mimicking the natural interaction observed during implantation. Yu et al. created a co-culture system using blastoids and immortalized primary endometrial stromal cells, though without the addition of epithelial cells [11]. Their work highlighted a role for endometrial stromal cells in promoting trophoblast syncytialization.

**Figure 2:**
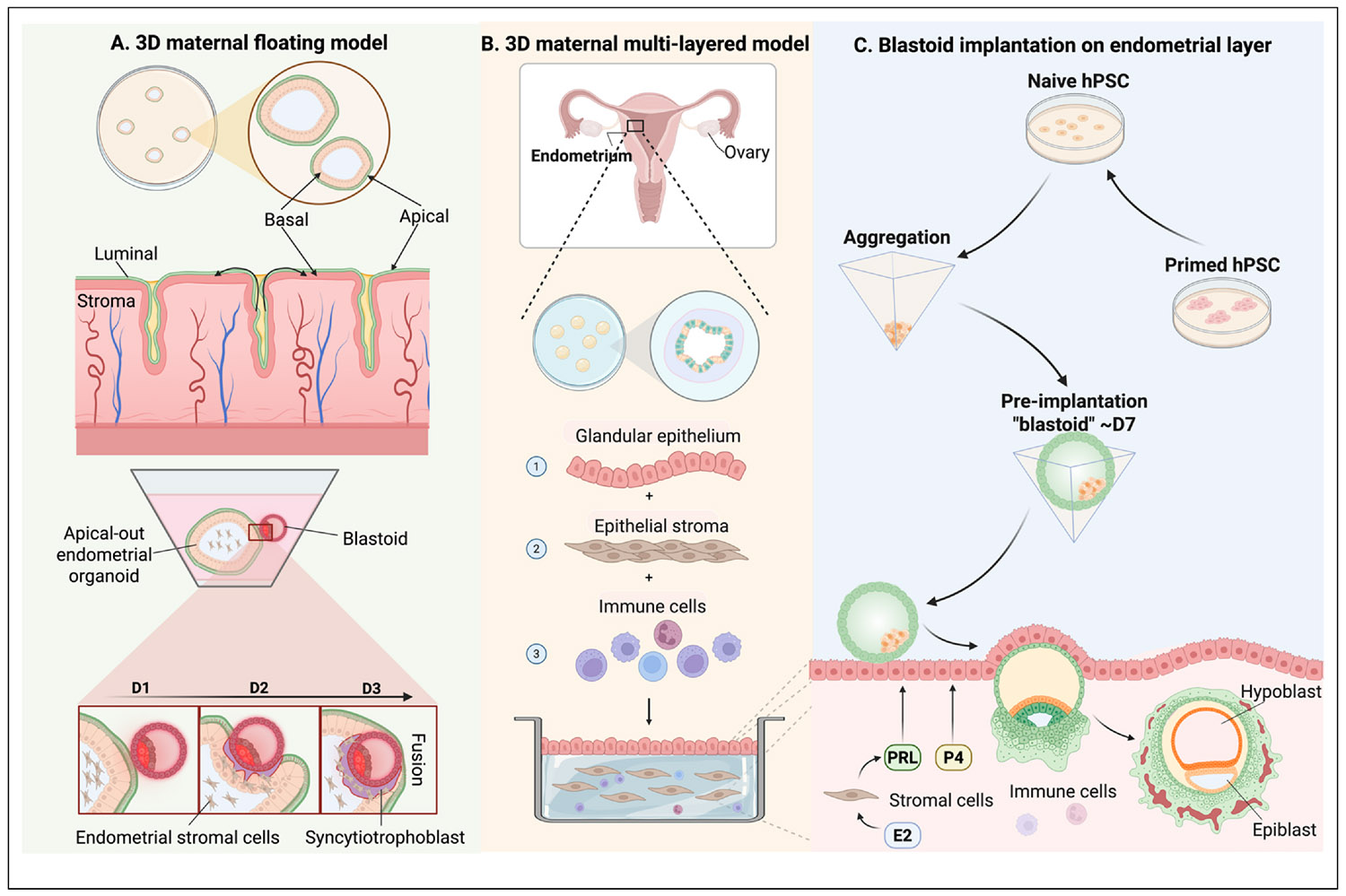
Stem cell–based models of human embryo implantation. **(a)** Shibata et al. [31] developed an AO-EMO system, which exposes the apical surface of the epithelium and incorporates stromal cells and an endothelial network. By co-culturing AO-EMOs with blastoids in a floating culture system, Shibata et al. were able to recapitulate key stages of implantation. **(b)** An alternative approach to building an *in vitro* endometrial model is to engineer an epithelial layer overlaying an appropriate ECM embedded with stromal fibroblasts, drawing on the concept of endometrial assembloids pioneered by Rawlings et al. [30]. This model can be further improved by incorporating immune and vascular cells. **(c)** By combining blastocyst-like structures (‘blastoids’) derived from naïve hPSC with the multilayered endometrial model described in (b), it will be possible to model human blastocyst apposition, adhesion, and invasion.

While the above approaches provide a first glimpse into maternal–fetal interactions, incorporating epithelial and stromal layers together will create a more comprehensive model for studying implantation and maternal–fetal communication. Rawlings et al. introduced the concept of endometrial ‘assembloids’ by combining primary stromal cells with epithelial organoids. When treated with hormones, these assembloids mimic gene expression changes typical of the mid-luteal phase. This coculture model highlights the influence of stromal cells on the epithelial environment, which is essential for creating a receptive endometrial lining during implantation [30]. Shibata et al. developed an apical-out endometrial organoid (AO-EMO) system, which exposes the apical surface of the epithelium and incorporates stromal cells and an endothelial network, thus closely emulating *in vivo* endometrial structure. By co-culturing these AO-EMOs with blastoids, Shibata et al. created feto-maternal assembloids that recapitulate key stages of implantation. This model provided insights into how syncytial trophoblasts disrupt the epithelial barrier and interact with stromal cells. Shibata et al. demonstrated that syncytial cells from the blastoids invade and fuse with endometrial stromal cells [31] (Figure 2a).

### Challenges and next steps for modeling human embryo implantation

Recent years have seen exciting progress in modeling human implantation, but studies so far have only examined blastoid development for a few days and lacked endometrial complexity. No research group has yet developed an apical-out maternal–fetal co-culture system integrating all essential cell types — trophoblast, endometrial, immune, and vascular — into a single model. A key challenge is incorporating endometrial vasculature, crucial for implantation success. Proper vascular remodeling enables low-resistance blood vessels essential for placental oxygen and nutrient supply [32]. Inadequate trophoblast-endometrial signaling can lead to vascular defects and implantation failure [33,34]. Another critical component at the maternal–fetal interface are immune cells, particularly decidual natural killer cells, which support immune adaptation for the semi-allogeneic embryo. Disruptions in immune tolerance can create an inhospitable implantation environment, increasing the risk of failure [35].

An important priority for the field is to develop a more sophisticated multilayered *in vitro* endometrial model that comprises an epithelial layer overlying an appropriate ECM embedded with stromal fibroblasts, immune, and vascular cells. Integrating human blastoids within such an endometrial assembloid will establish a more physiologically relevant platform for investigating maternal–fetal interactions (Figure 2b,c). An important challenge is to identify suitable culture media that can support the survival, proliferation, and development of these diverse cell types simultaneously. Another major issue is the reversal of apicobasal polarity in endometrial organoids since the apical side (facing the uterine cavity *in vivo*) faces inward in traditional organoid cultures. This orientation poses a problem for modeling maternal–fetal interactions, as it limits direct access to the cell surface where trophoblasts would naturally attach. Reversing this polarity to expose the apical side, including structures like microvilli, is essential for simulating the correct interactions at the maternal–fetal interface. However, achieving and maintaining this polarity in a controlled, scalable manner is technically challenging and requires further innovation in organoid and microfluidic culture techniques [34].

Implantation failure is thought to arise from disrupted communication between trophoblast cells and the maternal endometrium, resulting in impaired placental development, immune tolerance, and vascular remodeling [36]. Leveraging scRNA-seq and computational approaches could provide insights into the complex cellular interactions between blastoids and human endometrial assembloids. These studies can draw inspiration from single-cell explorations of ligand–receptor interactions at the human maternal–fetal interface [37]. By introducing targeted genetic edits in hPSCs before their differentiation into blastoids or endometrial lineages, it will be possible to assess the functional relevance of specific ligand–receptor interactions in blastoid implantation assays *in vitro*. Combining blastoids with patient-derived endometrial tissues [38] will enable investigation of the genetic origins of recurrent implantation failure.

## Human trophoblast invasion

### Current understanding and model systems

**Figure 3:**
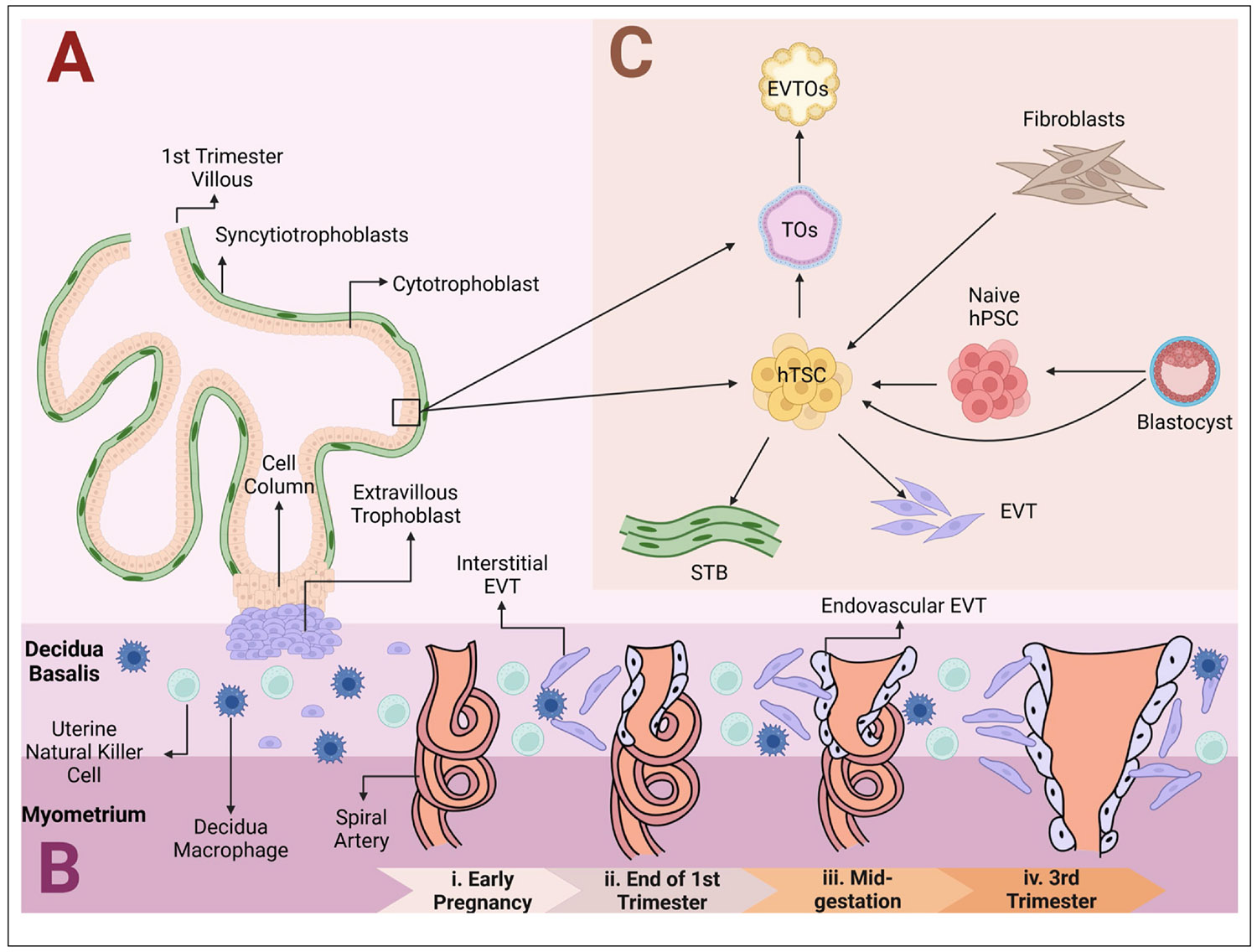
Stem cell–based models of human trophoblast invasion. **(a)** First-trimester primary villous tree highlighting the various trophoblast cell types present in the placenta. **(b)** Sequential steps in spiral artery remodeling as facilitated by EVTs and maternal immune cells from early pregnancy to the third trimester. **(c)** Current stem cell models developed to study placental development. hTSCs derived from first-trimester placental tissues, blastocysts, or naïve hPSCs can give rise to EVTs, STBs, and SC-TOs. The latter can further differentiate into invasive EVT organoids. This figure was adapted from references [65-67].

After implantation has occurred, the first trophoblast cells that invade into the maternal decidua are multi-nucleated primitive syncytial cells that form a conduit of nutrient and gas exchange with endometrial glands and maternal endothelial capillary beds [39-41]. Subsequently, cytotrophoblasts cells (CTBs) derived from TE rapidly differentiate into two functionally distinct terminally differentiated trophoblast cell types: the multi-nucleated, hormone-secreting syncytiotrophoblast (STB) cells and invasive extravillous trophoblast cells (EVT) [42] (Figure 3a). EVTs arise from the tips of villi that form a column extending to the maternal endometrium. EVTs eventually travel to the endometrium, complete a partial epithelial-to-mesenchymal transition [43], and invade multiple uterine structures [44]. EVT invasion into the decidua and myometrium begins around 17–18 days post-fertilization [45] with significant EVT presence in the decidua basalis by week 10 and deeper myometrial invasion by week 15 [46]. EVTs invade the decidua toward the spiral arteries and destroy the smooth muscle of the arteries, transforming them into high-conductance vessels [45] (Figure 3b).

Abnormalities in EVT invasion are a major contributor to poor placentation and pathological pregnancies with adverse effects on maternal health, fetal growth, and the timing of labor. Possible pathological outcomes include preeclampsia, placenta previa or accreta, fetal growth restriction, and early pregnancy loss [47]. Preeclampsia is thought to result from shallow trophoblast invasion into the maternal decidua. This poor invasion also results in improper remodeling of the spiral arteries, which leads to the vessels having high resistance to vascular flow. This in turn causes poor maternal–fetal circulation and dangerously high maternal blood pressure. Mothers who experienced preeclampsia are at a greater risk of contracting type 2 diabetes and cardiovascular problems postpregnancy. Currently, the only available treatment option for severe preeclampsia is the removal of the placenta through early delivery [47].

Given the aforementioned differences between mouse and human placentation (Figure 1), human-specific model systems are imperative to accurately investigate human placental development. In 2018, Okae et al. successfully derived hTSC from primary villous CTBs and blastocysts [13] (Figure 3c). To aid in their endeavor, Okae et al. analyzed transcriptomes of primary trophoblast cells to understand how CTBs maintain their undifferentiated state *in vivo*. They then leveraged this knowledge to develop a medium cocktail to derive hTSCs, which includes transforming growth factor beta (TGF-β) inhibitors, a histone deacetylase inhibitor, EGF, a Wnt activator, and a Rho-associated protein kinase (ROCK) inhibitor, collectively known as ‘SAVECY’. This culture medium allowed for the successful derivation of proliferative CTBs from first-trimester placental tissues. Importantly, they also demonstrated that these hTSC lines could differentiate into STB-like and EVT-like cells. Using the same culture conditions, Okae et al. were able to derive hTSCs from human blastocysts. Further characterization confirmed that these cells present the necessary hallmarks of bona fide hTSCs, including expression of placenta-specific protein markers and microRNAs, an appropriate HLA class I profile, and hypomethylation of the *ELF5* promoter [13].

While the derivation of hTSC was an important advance, limited access to first-trimester tissues and human blastocysts raised the question whether it may be possible to obtain similar hTSCs from stem cell sources. Three groups were able to generate hTSCs from hPSCs by first resetting conventional (also known as ‘primed’ hPSCs) into the naïve state, and subsequently applying SAVECY media [16-18]. In addition to using naïve hPSCs, adult fibroblasts can be reprogrammed and converted into hTSCs [18,48]. Transcriptome profiling showed that naïve hPSC-derived hTSCs and induced hTSCs shared the strongest transcriptional correlation to postimplantation trophoblast cells at 10–12 days post-fertilization. Importantly, by transiently treating naïve hPSCs with TGF-β and MEK/ERK inhibitors, Guo et al. and Io et al. were able to capture a preimplantation TE-like state before transitioning the cells into a postimplantation CTB state [49,50]. Thus, naïve hPSCs recapitulate the full arc of trophoblast differentiation from preimplantation TE to postimplantation CTB and specialized trophoblast lineages. Several groups have reported that primed hPSCs can also give rise to hTSCs, but the properties of hTSCs sourced from naïve and primed hPSCs require more careful examination. For example, recent evidence suggests that primed hPSC-derived hTSCs exhibit lower levels of placenta-specific miRNAs and reduced EVT invasion [51,52].

To better model *in vivo* placental development, *in vitro* systems that recapitulate the diverse cell types within the trophoblast stem cell niche are imperative. Developing 3D placental villus-like structures could bridge this gap. Turco et al. successfully isolated CTBs from first-trimester placental tissue and cultured them within Matrigel droplets to facilitate TO formation [14]. For this purpose, they developed a basal trophoblast organoid medium containing EGF, fibroblast growth factor 2 (FGF2), a TGF-β inhibitor, and two WNT agonists. To enhance cell viability, hepatocyte growth factor (HGF), prostaglandin, and a ROCK inhibitor were also added. Characterization of these organoids revealed that they develop into complex structures resembling the villous placenta both structurally and phenotypically. Haider et al. followed a similar protocol but opted to include a BMP inhibitor, while omitting HGF and FGF2 [15]. The TOs generated by both research groups can be converted into invasive EVT organoids by reducing the level of WNT signaling.

Restrictions on access to first-trimester placental tissues present a challenge to many researchers interested in modeling early placental development. The successful derivation of TOs from hTSCs has provided a more accessible 3D model of the early placenta. By aggregating naïve hPSC-derived hTSCs in Matrigel droplets, Karvas et al. established stem-cell-derived trophoblast organoids (SC-TOs) with comparable tissue architecture, placental hormone secretion, and long-term self-renewal as primary TOs [19]. Single-cell transcriptome profiling indicated that SC-TOs reflect the cellular diversity of trophoblast identities found in the human postimplantation embryo. Like primary TOs, these SC-TOs were able to differentiate into EVT organoids upon reduction of WNT signaling and showed robust invasion in a co-culture assay with human endometrial cells. SC-TOs were subject to X chromosome inactivation and had the ability to model selective vulnerability to emerging pathogens, validating their efficacy as a model of human placental development and disease [19].

### Challenges and next steps for modeling human trophoblast invasion

Currently available models of human trophoblast invasion face several challenges. First, there remains a significant debate whether the SAVECY conditions fully model hTSCs *in vivo* or represent a villous column progenitor that is biased toward EVT fate [53,54]. While this is not necessarily problematic for modeling trophoblast invasion, it does raise important questions when modeling trophoblast differentiation. For example, Shannon et al. conducted comparative scRNA-seq analysis on primary TOs, SC-TOs, and first-trimester placental tissues *in vivo* [55]. This work indicated that both primary TOs and SC-TOs produce the major trophoblast lineages found in placental tissues *in vivo*. However, primary TOs better reflected *in vivo* differentiation trajectories, largely due to the presence of a latent hTSC progenitor state within SC-TOs, which shares CTB and EVT characteristics. Despite these shortcomings, SC-TOs offer distinct advantages compared to primary TOs, given that they can be accessed from stem cell sources that are amenable to genetic engineering.

While hTSCs and TOs recapitulate EVT differentiation, currently available culture systems are unable to generate specialized EVT subtypes, such as endovascular trophoblast cells (eEVTs), interstitial EVTs (iEVTs), and placental bed giant cells (GCs). eEVTs form a plug close to the CTB shell where the maternal spiral arteries terminate, eventually replacing the endothelium, and transforming the arteries into high-conductance vessels [56]. iEVT cells are responsible for deep invasion into the myometrium to aid in attaching the placenta to the uterus [57,58]. Additionally, iEVTs interact with maternal immune cells and invade uterine veins [59]. The fusion of iEVTs into GCs within the inner third of the myometrium anchors the placenta to the uterus [60]. It has been speculated that the absence of maternal cells could explain why these specialized EVT lineages are missing in currently available *in vitro* trophoblast models. The recent identification of transcription factors associated with distinct EVT subtypes may guide the generation of more specialized EVT lineages *in vitro* [54].

When studying the driving forces behind EVT invasion, much importance has been attributed to hormones and growth factors present during pregnancy. However, there are important biochemical and biophysical cues that EVTs receive from the ECM, which is composed of fibrous proteins and viscous proteoglycans that create a 3D scaffold for cell adhesion [61]. The ECM also facilitates communication between the cells and their microenvironment to regulate migration and invasion while maintaining the structural integrity of the uterus during pregnancy. To help maintain these processes, the ECM attaches to trophoblast cells via membrane-bound adhesion molecules, in particular, integrins and selectins. Through these connections, trophoblast cells sense the physical characteristics of their microenvironment, including stiffness, pressure, shear, and stretch [61]. The typical ECM used for trophoblast studies is Matrigel, a commercially made gel-like derivative of a spontaneous tumor found in mice called the Engelbreth-Holm-Swarm tumor. This tumor is rich in basement membranes such as collagen and laminin, as well as other growth factors [62]. While Matrigel has been effectively used to culture human TOs, it displays high batch-to-batch variability. Matrigel is also too compliant, and its stiffness does not match that of the human decidua [63]. Therefore, a major challenge for future studies of human trophoblast invasion is to develop a biomimetic matrix from well-defined biomaterials that can replicate the mechanical properties of the receptive endometrium [64].

## Discussion

In recent years, significant progress has been made in developing novel model systems of human embryo implantation and trophoblast invasion. Pregnancy-related pathologies, such as recurrent implantation failure and preeclampsia, have severe impacts on maternal and fetal health. In this review, we discussed how novel stem cell–based models of the human embryo and placenta offer complementary insights into the sequential processes of human embryo implantation and trophoblast invasion. Blastoids generated from naïve hPSCs represent an integrated embryo model of the preimplantation blastocyst that captures hallmarks of early postimplantation development when cultured on appropriate ECM substrates. Importantly, blastoids faithfully model the transition from preimplantation TE into postimplantation trophoblast lineages. Consequently, blastoids provide a suitable model of human embryo implantation when combined with endometrial tissues obtained from patients or healthy volunteers. However, current ethical and technical restrictions limit blastoid culture to the first 2–3 weeks of human development. Since deeper myometrial invasion is not attained until week 15, nonintegrated embryo models that specifically model postimplantation trophoblast lineages are needed to study later stages of placentation. We propose that this knowledge gap can be addressed using hTSCs, which have been derived from a wide range of sources, including blastocysts, first-trimester placental tissues, naïve hPSCs, and somatic cells. Because hTSCs can differentiate into specialized trophoblast lineages and organoids, they offer an accessible and ethically acceptable *in vitro* model of human trophoblast differentiation and invasion. Nevertheless, important challenges need to be overcome to realize their full potential as a model of human placental development. These challenges include refining the culture conditions to capture hTSCs that more closely resemble bipotent hTSCs *in vivo*, generating more specialized EVT lineages, and engineering a biomimetic decidua-like ECM that recapitulates the biomechanical properties of the receptive endometrium. The advent of these stem cell–based embryo and placenta models heralds an exciting era for studies of human embryo implantation and trophoblast invasion.

## Data Availability

No data were used for the research described in the article.
